# Relation of Physical Activity Level to Postural Balance in Obese and Overweight Spanish Adult Males: A Cross-Sectional Study

**DOI:** 10.3390/ijerph18168282

**Published:** 2021-08-05

**Authors:** José Manuel Delfa-de la Morena, Eliane A. Castro, Miguel Ángel Rojo-Tirado, Daniel Bores-García

**Affiliations:** 1 Department of Physical Therapy, Occupational Therapy, Rehabilitation and Physical Medicine, Rey Juan Carlos University of Madrid, Avenida de Atenas, s/n, 28922 Madrid, Spain; jose.delfa@urjc.es; 2 LFE Research Group, Department of Health and Human Performance, Faculty of Physical Activity and Sport Science (INEF), Universidad Politécnica de Madrid, 28040 Madrid, Spain; eliane.castro@alumnos.upm.es (E.A.C.); ma.rojo@upm.es (M.Á.R.-T.); 3 Department of Sports Sciences and Physical Conditioning, Faculty of Education, Universidad Católica de la Santísima Concepción, Concepción 4070129, Chile

**Keywords:** sedentary behavior, obesity, adipose tissue, sensory organization test

## Abstract

The aim of this study was to analyze the influence of physical activity level on postural control in obese and overweight Spanish adult males. Forty-three males aged between 25 and 60 years old were included. Anthropometric, body composition, and physical activity variables were assessed, and postural control was evaluated using the Sensory Organization Test. No correlation was found between the level of physical activity and postural control, assessed by the Sensory Organization Test within the whole sample. However, within the group with a higher total fat mass percentage, non-sedentary individuals presented improved scores on the somatosensory organization test when compared to sedentary individuals (96.9 ± 1.8 vs. 95.4 ± 1.2; *p* < 0.05) and poorer scores on the composite equilibrium score (73.4 ± 7.2 vs. 79.2 ± 6.9; *p* < 0.05). The altered integration of somatosensory inputs most likely affects the tuning, sequencing, and execution of balance strategies in sedentary men with a high total fat mass percentage.

## 1. Introduction

Postural control is essential for the functional capacity of individuals. Activities of daily living such as walking, climbing stairs, getting up from a seated position or reaching objects during standing require a stable balance [1,2,3]. Most studies from the scientific literature have demonstrated that an active lifestyle improves postural control [4,5,6,7,8] and is a determinant factor in the prevention of falls [4,5,9,10,11,12,13,14]. Balance or postural stability is achieved by maintaining the center of body mass over the base of support provided by the feet. To maintain balance, postural control integrates visual, vestibular and somatosensory (mechanoreceptive and proprioceptive [14]) information in order to generate appropriate motor responses [4,15,16]. Previous studies have reported that increased weight [17,18] and higher body fat mass percentage [19,20,21] can affect postural control, increasing the propagation forces of plantar shear, especially in dynamic tasks [22,23,24,25,26], and modifying changing body geometry [27], so tending to place the center of mass of the whole body further forward [22]. Obesity and a sedentary lifestyle have become the factors that most affect balance [27,28,29,30,31,32,33], increasing the risk of falls [17,34,35,36,37] and lower limb injuries [38,39,40] as well as causing reduced plantar cutaneous mechanoreceptor sensitivity [41] and impaired gait mechanics, [42] especially in obese males [43,44], being related to the alteration of biomechanics and plantar load that arises from a chronic increase in body mass [22]. Moreover, obesity has been related to increased ankle muscle activity to counteract gravitational torque [17] and/or a deficiency in sensory integration processes during postural control tasks [45]. In fact, a higher sway velocity and a greater sway amplitude during upright standing in obese subjects compared to normal weight subjects have previously been shown in the scientific literature [46], determining that obese individuals spend less time in zones of stability and the distance between these zones of stability is greater, suggesting that there are difficulties in controlling balance even with an intact sensory system. Previous studies have demonstrated improved balance in obese individuals after a weight loss and physical activity program [47], probably by improving their muscular and proprioceptive systems that may induce a better functional capacity, quality of life and independence [27]. Furthermore, the 2018 Physical Activity Guidelines Advisory Committee stated that physical exercise improves physical function and reduces the risk of falls and fall-related injuries [48], in agreement with previous studies that suggest that strength and balance training can improve postural control capacity in obese elderly people [19,49]. However, to our knowledge, no studies have described the relationship of the physical activity level with postural control in overweight and obese adult men. Thus, this study sought to analyze the influence of a sedentary or non-sedentary lifestyle on postural balance in Spanish adult men with overweight and obesity.

## 2. Materials and Methods

### 2.1. Design

An observational, cross-sectional, descriptive study was performed using non-probabilistic consecutive sampling.

### 2.2. Participants

Volunteers were recruited via email from the Nutritional and Physical Activity Program for the Control of Obesity project (PRONAF, ClinicalTrials.gov Identifier: NCT01116856) [50]. From the total of participants of the PRONAF project, 131 individuals expressed interest to participate in this study. Forty-six males aged between 25 and 60 years old, with a BMI between 18 and 35 kg/m^2^, a stable body weight (no weight gain or loss of 2 kg or more during the past 3 months), and with a level of physical activity classified as sedentary or low active: PAL < 1.6 [51], measured via accelerometry, were included in the study. Participants suffering from serious illnesses, smokers, recent ex-smokers (abstinent for less than 6 months), consumers of alcohol, participants diagnosed with balance disorders, participants with knee or hip replacements, those suffering from arthritis or other severe inflammatory diseases affecting the lower limbs, or those who had suffered from trauma to the lower limbs in the previous 6 months were excluded from the study. During data collection, 3 participants dropped out due to personal motives. Finally, 43 participants completed the study. All participants were provided with written information detailing the nature and purpose of the study. The protocol was approved by the institutional ethics committee of the University Rey Juan Carlos and was in accordance with the Declaration of Helsinki for Human Research.

### 2.3. Measurements

Anthropometric variables. Weight (kg) was measured using a TANITA BC-420MA balance scale (Bio Lógica Tecnología Médica S.L, Barcelona, Spain), and height (m) with a SECA stadiometer (range 80–200 cm, Valencia, Spain). From these measurements, Body Mass Index (BMI, kg/m^2^) was calculated. Body composition was assessed by Dual X-ray absorptiometry (DXA) [35,52] using a GE Lunar Prodigy densitometer (GE Healthcare, Madison, Wisconsin, USA). Body composition variables were total fat mass percentage and total lean mass percentage. The median of total fat mass percentage (32.9%) was calculated in order to classify the participants into either low or high fat mass percentage and enable the comparison of results within each group in function of physical activity level in accordance with the Food and Nutrition Board of the Institute of Medicine [51].

Physical activity was assessed by accelerometry [53] using a SenseWear Armband Pro 3 accelerometer (SWA, BodyMedia Inc., Pittsburgh, Pennsylvania). The SWA features a 2-axis accelerometer, heat flux sensor, galvanic skin response sensor, skin temperature sensor, and a near-body ambient temperature sensor [54], which provides information about calories burned, steps taken and minutes spent in moderate and vigorous physical activity [55]. Participants wore SWA on the back of their dominant upper arm for approximately 7 days to measure energy expenditure connected to physical activity [56]. Participants were classified in sedentary or non-sedentary according to Physical Activity Level (PAL) parameter. PAL value of 1.4 was considered, with men with higher values grouped as “non-sedentary” (low active) and men with lower or equal values grouped as “sedentary” (inactive) [53].

Postural control was assessed via posturography [18,57]. Specifically, balance was assessed by the Sensory Organization Test (SOT), using the SMART EquiTest^®^ computerized dynamic posturographic system (Neurocom International, Clackamas, Oreg., USA). This apparatus consists of a force platform and a visual surround that can be either fixed or mobile (the system rotates around the ankle joints in response to the individual’s postural adjustments). The SOT can be used with the eyes open or closed, and provides the individual with information on a somatosensory, visual, and vestibular level. Six different test conditions were used: (1) eyes open, visual surround and fixed support; (2) eyes closed, fixed support; (3) mobile visual surround and fixed support; (4) fixed visual surround and mobile support; (5) eyes closed, mobile support; and (6) eyes open, visual surround and mobile support. Three 20-s measurements were taken in each condition. The SMART EquiTest^®^ is described in detail elsewhere [58]. Based on these 6 conditions, the values of the SOT test were obtained to quantify the Composite Equilibrium Score (SOT-CES): the global ability of the subject to maintain balance; the somatosensory organization test (SOT-SOM): the ability of the subject to use the somatosensory stimulus to maintain balance; the visual sensory organization test (SOT-VIS): the ability of the subject to use the visual stimulus to maintain balance; the vestibular sensory organization test (SOT-VEST): the ability of the subject to use input from the vestibular system to maintain balance; and the preferential sensory organization test (SOT-PREF): the degree to which a subject relies on the visual information to maintain balance, even when the information is incorrect.

### 2.4. Statistical Analysis

The statistical analysis of the data was carried out using the Statistical Program for Social Science (SPSS) version 17 (SPSS Inc., Chicago, Illinois, USA). The Shapiro–Wilk test was used to test the normality of the data. Description variables presented normal distribution; therefore, unpaired Student’s t-tests were performed for comparing between individuals classified as sedentary or non-sedentary. For balance variables that did not present normal distribution, the Mann–Whitney U test was used for the same comparison. The significance level was set at α < 0.05. Results are presented as mean ± standard deviation.

## 3. Results

The 43 Spanish male participants were divided into sedentary and non-sedentary. Significant differences between sedentary and non-sedentary participants were found for body weight (94.6 ± 7.7 kg vs. 88.1 ± 10.0 kg, *p* = 0.038), total fat mass percentage (35.0 ± 5.3% vs. 29.6 ± 5.8%, *p* = 0.005) and total lean mass percentage (62.6 ± 5.1% vs. 67.9 ± 5.8%, *p* = 0.005). The characteristics of the study sample are shown in Table 1.

**Figure 1:**
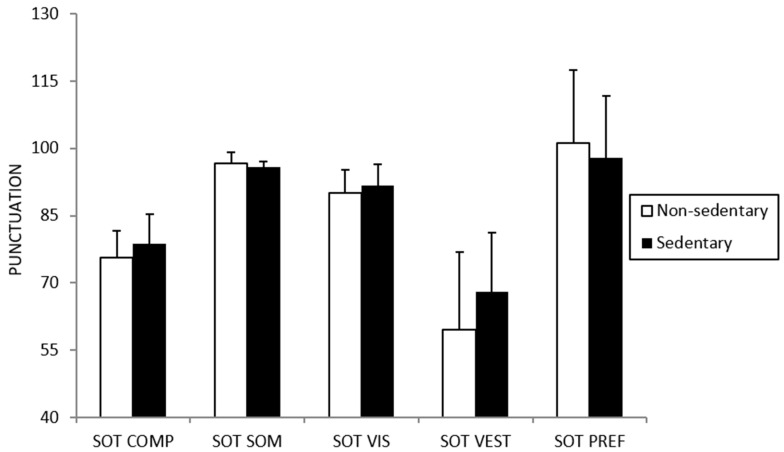
Differences in postural control between sedentary and non-sedentary participants.

No differences were found between sedentary and non-sedentary participants for the variables related to postural control (Figure 1). A tendency was only found for the SOT-CES (SOT-COMP in Figure 1) component (*p* = 0.061), with greater values for the sedentary participants.

There were differences between sedentary and non-sedentary participants when the median of the total fat mass percentage was considered (see Table 2). Non-sedentary participants with a high total fat mass percentage presented better results in the SOT-SOM and worse results in the SOT-CES compared to their sedentary counterparts (i.e., 96.9 ± 1.8 vs. 95.4 ± 1.2, *p* = 0.03; 73.4 ± 7.2 vs. 79.2 ± 6.9, *p* = 0.02, respectively). On the other hand, no significant differences were found between sedentary and non-sedentary participants with a low total fat mass percentage.

## 4. Discussion

Many authors have recognized that an active lifestyle contributes to improved postural stability [1,3,4,5]. Contrary to that, we found no correlation between physical activity level and postural control, assessed using the SOT. Our results could be explained mainly by the fact that most of the samples of the previously cited studies investigated postural control in normal weight or overweight participants, while the population of the present study consisted of adult male participants who were overweight or obese [28]. Our findings are in agreement with Stemplezki et al. [59], who reported that the level of physical activity did not influence postural control in a sample of 17 overweight and obese adults. In addition, the higher BMI values of our sample may be interfering with the results of the effect of physical activity on balance. In adulthood, when comparing participants with different BMIs, it is important to note that BMI is mainly related to body mass variations because height remains stable. Thus, higher BMI values are due to excess body mass. Generally, this increased weight is associated with an increase in fat mass and concomitant decreased physical activity [60]. Indeed, several authors have reported that a higher fat mass percentage is negatively related to postural stability [20,21,30,61,62]. This relationship is more marked in males [34,43,44] due to accumulated fat mass in the abdominal region. Considering an inverted pendulum, this fat mass causes a displacement of the center of mass in the anteroposterior direction, and consequently, more difficulty in maintaining balance [63].

Within the group with a high total fat mass percentage, our results revealed significant differences in postural control between sedentary and non-sedentary individuals. Specifically, non-sedentary individuals were characterized by greater SOT-SOM scores than their sedentary counterparts, as well as poorer SOT-CES scores. These results may be due to the different perceptual–sensory processes implicated in postural control. Since these processes are visual, vestibular and somatosensory (mechanoreceptors and proprioceptors) systems [4,15,16], the integration of these sensory inputs provides accurate information to the central nervous system about how the body is orientated (internally and externally) and whether the body is stationary or moving. Among these three sensory inputs, visual input is the one humans rely on the most, but without it, the proprioceptive system becomes the main source of sensitive information to maintain balance both while standing and moving [64,65]. The SOT-SOM scores are calculated on a stable surface when visual cues are removed (eyes closed). Abandoning physical activity and adopting a sedentary lifestyle probably reduces sensory stimulation, especially of both vestibular and proprioceptive receptors [4], so it is understood that a non-sedentary lifestyle increases the sensory stimulation of these receptors. In this line, Islam et al. [66] found a positive correlation between habitual physical activity (measured by an accelerometer) and the ability to maintain balance on one leg with eyes closed. In addition, recent studies have revealed that participants who practice some physical activity, such as skating or taekwondo, presented greater SOT-SOM scores when compared to their sedentary counterparts [3,67], indicating that physical activity practitioners develop and rely more on their proprioceptive system for maintaining balance.

Furthermore, an excess fat mass is associated with a deterioration in the quality of both muscles and joints, inducing a decrease in the efficiency of the proprioceptive system [68]. This deterioration induced by excess fat mass could be partially compensated in non-sedentary participants by stimuli implied by physical activity [69]. In a study involving elderly people with no sensory conflicts under normal conditions, Lord et al. [70] reported that postural sway was related to the deterioration of tactile receptors, towards joint positions and decreased reaction time, but not due to decreased vestibular or visual function or muscular strength. This is the reason why the authors suggest that the proprioceptive system is the most important sensory system for maintaining balance on a stable surface. In the same line, Nardone et al. [71] state that proprioception and sensory stimulation of the plantar surface of the foot are considered the main sensory systems for maintaining postural stability in normal conditions. Somatosensory inputs may be deteriorated in sedentary individuals with excess fat mass, and to a lesser extent in obese non-sedentary individuals because of physical activity. This mainly explains the higher SOT-SOM scores found in non-sedentary individuals with a high fat mass percentage compared to their sedentary counterparts.

On the other hand, our results showed higher SOT-CES scores among sedentary individuals with a high fat mass percentage compared to their non-sedentary counterparts. As indicated earlier, non-sedentary individuals likely rely on their proprioceptive system for maintaining balance. Information from somatosensory afferents is inaccurate in most conditions that are considered to calculate SOT-CES scores. This could explain the poorer SOT-CES scores of our non-sedentary individuals compared to their sedentary counterparts. Moreover, it is also interesting to highlight the fact that in situations where visual and proprioceptive systems are compromised (as mainly for SOT-CES), greater postural sway will not always be related to worse postural control but may be due to intentional movements to obtain the best available sensory information. In other words, changes in postural sway when information via visual and/or somatosensory systems is inaccurate could reflect the most appropriate postural control strategy in these conditions [72]. These facts could try to explain why our non-sedentary individuals, who rely more on the somatosensory system, obtained worse SOT-CES scores than their sedentary counterparts.

This study presents some limitations, such as the absence of a control group with normal weight individuals and a relatively small sample size. On the other hand, this work also has several strengths as it provides a relevant assessment of the influence of the physical activity level (assessed by accelerometry) on the postural control (evaluated by posturography) of men with excess fat mass (assessed by dual-energy X-ray absorptiometry). As a future line of research, it would be interesting to conduct longitudinal studies with obese children and adolescents, following them throughout life. Additionally, it would be interesting to study different types of physical activities, including work and leisure activities.

## 5. Conclusions

In conclusion, our study demonstrated that in adult males with a high total fat mass percentage, postural stability is maintained if a non-sedentary lifestyle is adopted. This was most likely due to the effective integration of somatosensory inputs. Thus, physical activity may, at least in part, counteract the deterioration in the efficiency of somatosensory inputs on imbalance produced by excess fat mass. Our findings may be useful for planning physical activity programs for the health promotion of the obese population, trying to counteract or, at least, delay the age-typical loss of balance with aging [22,27,33,45], because aging causes progressive changes in the neuromuscular, proprioceptive, and visual systems, as well as in sensory integration [73,74,75,76]. Further research is needed to understand the relation between physical activity and body composition with postural control.

## Figures and Tables

**Table 1:** Characteristics of the study sample (*n* = 43).

Variables	Sedentary (*n* = 14)	Non-Sedentary (*n* = 29)
Age (years)	43.86 ± 5.27	42.07 ± 6.01
Weight (kg)	94.57 ± 7.68	88.09 ^a^ ± 9.96
Height (m)	176.25 ± 6.89	173.79 ± 6.03
Body Mass Index (kg/m^2^)	30.46 ± 2.03	29.13 ± 2.59
Total Fat Mass (%)	35.01 ± 5.34	29.60 ^a^ ± 5.80
Total Lean Mass (%)	62.61 ± 5.15	67.95 ^a^ ± 5.78
Physical Activity Level	1.28 ± 0.05	1.54 ^b^ ± 0.12

Note. Data are presented as mean ± SD. ^a^ *p* < 0.05; ^b^ *p* < 0.001; differences between sedentary and non-sedentary subjects.

**Table 2:** Comparison of the postural control tests in individuals with a low or high total fat mass percentage.

	High Percentage (≥32.9%)			Low Percentage (<32.9%)		
	Sedentary (*n* = 10)	Non-Sedentary (*n* = 11)	*p*-Value	Sedentary (*n* = 4)	Non-Sedentary (*n* = 18)	*p*-Value
SOT CES	79.20 ± 6.94	73.45 ^a^ ± 7.17	0.020	77.50 ± 6.76	76.89 ± 4.81	0.902
SOT SOM	95.40 ± 1.17	96.91 ^a^ ± 1.76	0.029	97.00 ± 0.82	96.44 ± 2.93	0.967
SOT VIS	92.70 ± 2.63	90.09 ± 3.75	0.132	89.25 ± 8.30	90.06 ± 5.83	0.999
SOT VEST	69.60 ± 10.56	53.27 ± 23.34	0.132	63.75 ± 20.07	63.28 ± 11.89	0.967
SOT PREF	96.50 ± 12.99	103.18 ± 24.24	0.756	101.25 ± 17.75	100.00 ± 9.44	0.594

Note. SOT-CES: composite equilibrium score; SOT-SOM: somatosensory organization test; SOT-VIS: visual sensory organization test; SOT-VEST: vestibular sensory organization test; SOT-PREF: preferential sensory organization test. ^a^ Differences between sedentary and non-sedentary subjects, *p* < 0.05.
